# Differential expression of TgMIC1 in isolates of Chinese 1 *Toxoplasma* with different virulence

**DOI:** 10.1186/s13071-021-04752-z

**Published:** 2021-05-13

**Authors:** Yang Wang, Chengjian Han, Rongsheng zhou, Jinjin Zhu, Famin Zhang, Jingyang Li, Qingli Luo, Jian Du, Deyong Chu, Yihong Cai, Jilong Shen, Li Yu

**Affiliations:** 1 Department of Microbiology and Parasitology, Anhui Provincial Laboratory of Microbiology and Parasitology; Anhui Key Laboratory of Zoonoses, School of Basic Medical Sciences, Anhui Medical University, 81 Meishan Road, Hefei, 230032 Anhui Province People’s Republic of China; 2 The Clinical Laboratory of the Third People’s Hospital of Heifei, Hefei, China; 3 Department of Biochemistry and Molecular Biology, Anhui Medical University, Hefei, China; 4 Department of Health Inspection and Quarantine, School of Public Health, Anhui Medical University, Hefei, China

**Keywords:** *Toxoplasma gondii*, Invasion, Microneme protein 1, Polyclonal antibody

## Abstract

**Background:**

The predominant genotype of *Toxoplasma* in China is the Chinese 1 (ToxoDB#9) lineage. TgCtwh3 and TgCtwh6 are two representative strains of Chinese 1, exhibiting high and low virulence to mice, respectively. Little is known regarding the virulence mechanism of this non-classical genotype. Our previous RNA sequencing data revealed differential mRNA levels of TgMIC1 in TgCtwh3 and TgCtwh6. We aim to further confirm the differential expression of TgMIC1 and its significance in this atypical genotype.

**Methods:**

Quantitative real-time PCR was used to verify the RNA sequencing data; then, polyclonal antibodies against TgMIC1 were prepared and identified. Moreover, the invasion and proliferation of the parasite in HFF cells were observed after treatment with TgMIC1 polyclonal antibody or not.

**Results:**

The data showed that the protein level of TgMIC1 was significantly higher in high-virulence strain TgCtwh3 than in low-virulence strain TgCtwh6 and that the invasion and proliferation of TgCtwh3 were inhibited by TgMIC1 polyclonal antibody.

**Conclusion:**

Differential expression of TgMIC1 in TgCtwh3 and TgCtwh6 may explain, at least partly, the virulence mechanism of this atypical genotype.

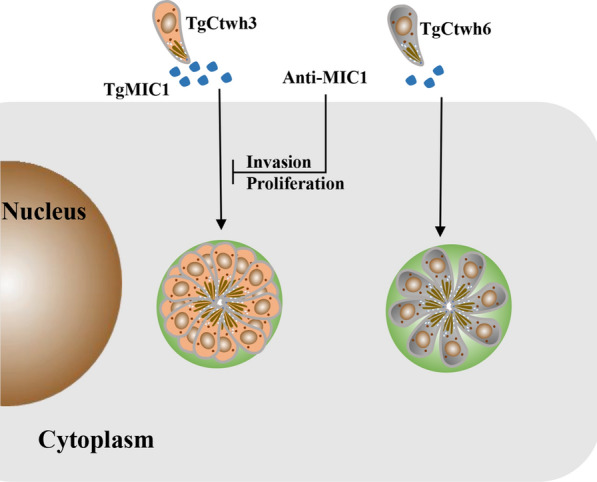

## Background

*Toxoplasma gondii* is an intracellular parasite belonging to the phylum Apicomplexa that infects a wide range of warm-blooded vertebrates, including approximately one-third of humans [1]. In most hosts, this intracellular protozoan parasite escapes immunological surveillance and can cross the blood-brain and blood-retina barrier reaching alleged immune privileged regions to establish a life-long latent infection [2]. The opportunistic characteristic of *T. gondii* has rendered most infected healthy individuals asymptomatic. However, in immunosuppressed or congenitally infected individuals, infection can cause encephalitis, ocular disease or even death and is of great clinical importance [3]. Therefore, a better understanding of the molecular mechanisms responsible for the differences in the virulence of *T. gondii* is needed to develop effective vaccines or drugs to control the spread of toxoplasmosis.

It is perhaps unsurprising that *T. gondii* subverts host protein networks depending on the infected cell type and the parasite genotype. Genomic differences in *T. gondii* strains (types I, II and III are the classical North American and European strains) are typically revealed by techniques such as restriction fragment length polymorphisms or isoenzyme markers [4]. Type I strains have a high level of acute virulence in laboratory mice, with an LD_100_ as low as a single tachyzoite. Although the type II strain has relatively reduced virulence, it is most commonly associated with human infections. Type III strain is avirulent and always exists in domestic and wild animals; it is rarely found in human infections [5]. Compared to the North American and European strains, South American strains show more complex and diverse genetic polymorphisms. More importantly, the predominant genotype of *T. gondii* in China is Chinese I (ToxoDB#9) lineage (> 79% based on 60 isolates) [6]. Furthermore, our previous studies have demonstrated that the isolates of Chinese I vary in their virulence in mice; among these, TgCtwh3 exhibits high virulence, whereas TgCtwh6 exhibits low virulence [6–8].

Upon infection, *T. gondii* tachyzoites are surrounded by a non-fusogenic compartment, termed the parasitophorous vacuole (PV), to survive in the host cytoplasm by avoiding lysosomal degradation [9, 10]. This active process depends on the discharge of parasite proteins from its specialized set of secretory organelles, namely micronemes, rhoptries and dense granules. Among these, proteins secreted from the micronemes (MICs) are involved in the initial recognition, attachment and invasion, whereas those secreted from the dense granules (GRAs) and rhoptries (ROPs) participate in modulating a variety of host signals to establish a suitable environment for parasite growth [11, 12]. Revealed by genetic manipulation, some TgMIC proteins are proved to be critical virulence factors including TgMIC1. Rather than relying on transmembrane (TM) domains or glycosylphosphatidylinositol (GPI) anchors, TgMIC1 contains two microneme adhesive repeat (MAR) domains that form a complex with TgMIC4/TgMIC6 to achieve surface localization [13, 14]. The critical role of TgMIC1 in parasite invasion and contributing to virulence in mice has been reported [15, 16]. Additionally, a recent study showed that TgMIC1 directly binds to the N-glycans of TLR2, thereby affecting systemic levels of IL-12 and IFN-γ *in vivo* [17]. In this study, we aim to describe the differential expression levels of the TgMIC1 gene in TgCtwh3 and TgCtwh6. Furthermore, given its influential role in *T. gondii* infection competency and murine pathogenesis, TgMIC1 could be a critical virulence factor between these two strains. To investigate this possibility, differences in TgMIC1 protein expression in TgCtwh3 and TgCtwh6 were evaluated using a polyclonal antibody.

## Methods

### Cells and parasites

Human foreskin fibroblast (HFF) cells were purchased from ATCC (SCRC-1041) and routinely maintained in Dulbecco’s Modified Eagle Medium (DMEM, Gibco) supplemented with 10% fetal bovine serum (FBS, BI, Israel), 100 µg/ml penicillin and 100 µg/ml streptomycin (Sigma, USA) and maintained in an incubator at 37 °C and 5% CO_2_. *Toxoplasma gondii* RH strain, green fluorescent protein-RH strain (GFP-RH), TgCtwh3 and TgCtwh6 were propagated in HFF cells.

### RNA extraction and quantitative real-time PCR assays

Total RNA was extracted from the parasites using Trizol Reagent (Invitrogen, USA). Total RNA was used for reverse transcription with the cDNA synthesis kit (TaKaRa, Japan) according to the manufacture’s protocol. cDNA synthesis was performed using SYBR-Green Master Mix (TaKaRa, Japan). The primer sequences used were as follows:

TgMIC1 forward, TCGGTTTATGCTGAGTGTGC;

TgMIC1 reverse, GGCGAATTCCTTCCTCTTCT;

TgMIC4 forward, GACATGACGGGATCCAGAAC;

TgMIC4 reverse, CATGCAACTTGGCAGTCTGT;

TgMIC6 forward, CATATCACCTGCAAGCGTGT;

TgMIC6 reverse, GGCTCACGACTTTCACCTTC;

β-tublin forward, GTCTCCACTTCTTCCTCATTG;

β-tublin reverse, GTTCTTTGCGTCGAACATC.

β-Tublin was used as an internal control. Relative expression levels were calculated according to the standard 2^−ΔΔC^t method. All experiments were performed in triplicate and repeated at least three times.

### Expression and purification of recombinant TgMIC1 proteins

The coding sequence (CDS) of TgMIC1 was optimized by using the Optigene™ codon optimized analysis platform (Shanghai Jierui Bioengineering Co., Ltd.). The optimized TgMIC1 sequence was synthesized and cloned into pET30a. The recombinant pET30a-MIC1 plasmid was transformed into *Escherichia coli* BL21 (DE3), cultivated in Luria-Bertani (LB) at 37 °C. The recombinant protein expression was induced by adding 0.5 mM isopropyl β-D-1-thiogalactopyranoside (IPTG, Sigma, USA) at 16 °C overnight with constant shaking at 200 rpm. Then, the bacterial cells were harvested by centrifugation at 8000 rpm for 6 min and resuspended in 20 ml 10 mM Tris–HCL buffer. The bacteria suspension was sonicated on ice (500 W, 180 times, 5 s each time, 5 s interval). The lysate was centrifuged at 12,000 rpm for 10 min to separate the supernatant and bacterial debris. The level of rTgMIC1 expression was analyzed by 12% SDS-PAGE. Furthermore, the separated supernatants were collected and purified by Ni column (Ni Sepharose 6 Fast Flow, GE Healthcare). The purification efficiency was analyzed via 12% SDS-PAGE and the protein concentration measured by BCA Protein Quantitation Kit according to the instruction's protocol.

### TgMIC1 polyclonal antibody preparation and specific identification

For polyclonal antibody production, three 2-kg New Zealand white rabbits were immunized with 400 µg of purified rTgMIC1 diluted in 200 µl phosphate-buffered saline (PBS) and mixed with an equal volume of complete Freund’s adjuvant (Sigma, USA), through multiple intradermal injections into the back of each rabbit. Two weeks later, the immunization was boosted with 200 µg rTgMIC1 protein in Freund's incomplete adjuvant followed by a booster immunization once every 2 weeks. The ear vein blood was collected 7 days after the fourth immunization to measure the titer of antibodies by enzyme-linked immunosorbent assay (ELISA). All rabbits were anesthetized by injecting 3% pentobarbital sodium (1 ml/kg) at the base of the ear prior to the terminal cardiac puncture. The rabbit heart was then bled, and the antibodies were purified by Protein A [18]. The serum was mixed with an equal volume of the binding buffer to equilibrate the column. After the serum sample was loaded, the column was rinsed with binding buffer until the binding solution contained no protein. The presence of TgMIC1 polyclonal antibody was further identified by western blot and immunofluorescence assays.

### Protein isolation and western blot assays

The rTgMIC1 and recombinant plasmid were prepared as described above. Parasites was lysed in the ice-cold RIPA lysis buffer (Beyotime, China) with 1% phenyl methyl sulfonyl fluoride, and the total protein concentrations were detected by BCA Protein Quantitation Kit. The proteins (20 µg) were subjected to electrophoresis in 8–10% polyacrylamide gel. The proteins were transferred onto a polyvinylidene fluoride membrane (Millipore, USA) by a standard western blot procedure. The membrane was blocked with 5% nonfat skim milk in TBS containing 0.1% Tween 20 for 1 h and incubated with specific primary antibodies against TgMIC1 (1:4000), Tgβ-actin (1:2000) or HIS monoclonal antibodies (1:5000) at 4 °C overnight. Following incubation with the corresponding secondary antibodies (Santa Cruz Biotechnology, 1:10,000 diluted to 5% nonfat skim milk) conjugated to horseradish peroxidase for 1 h, the membranes were visualized using ECL Western blotting substrate (Bio-RAD, USA). The band intensity was quantified using ImageJ software, and the TgMIC1 protein expression was normalized to the Tgβ-actin protein level.

### ELISA analysis

The titer in the serum from immunized rabbits was performed with ELISA. The purified rMIC1 protein (200 ng/ml) was used to coat the plate at 4 °C overnight, followed by blocking with 5% BSA at 37 °C for 60 min. The serum sample was serially diluted from 1:200 to 1:204,800, and then 100 µl of diluted sample was added to each well and incubated 60 min at 37 °C. The samples were further incubated with secondary anti-rabbit antibodies (Zhongshan Golden Bridge, Beijing, China) diluted 1:5000 in PBS conjugated to horseradish peroxidase in each well for 1 h. Then, the wells were incubated with 100 µl tetramethylbenzidine (TMB, Beyotime, China) at 37 °C for 10 min. Finally, 2 M H_2_SO_4_ (50 µl) was used to stop the reaction in the well, and the absorbance was read at 450 nm. All results were repeated three to four times independently.

### Immunofluorescence staining

HFF monolayers were grown on coverslips placed in wells of a 6-well plate at a density of 10^5^ cells per well and were incubated for 24 h. Then, cells were challenged with *T. gondii* GFP-RH strains for 24 h. After washing with PBS three times, the coverslips were fixed with 4% paraformaldehyde (PFA) for 20 min. The coverslips were permeabilized with 0.1% Triton X-100 (Sigma, USA) for 30 min, and blocked for 30 min at 37 °C with 5% BSA in PBS. After washing again, the coverslips were then incubated with polyclonal antibody against TgMIC1 (1:1,000) overnight at 4 °C, followed by goat anti-rabbit secondary antibodies conjugated to Alexa Fluor 594 (1:500, Invitrogen, USA). Images were recorded using a Zeiss LSM880 confocal microscope (Zeiss).

### Invasion assay

HFF monolayers were grown on coverslips placed in wells of a 6-well plate at a density of 10^5^ cells per well and were incubated for 24 h. First, cells were incubated with TgMIC1 polyclonal antibody at 37 °C for 16 h. Then, the cells were challenged with *T. gondii* TgCtwh3 and TgCtwh6 strains for 1 h. Non-adherent parasites were washed away with PBS before fixation with 4% PFA for 20 min. Adherent external parasites were detected using rabbit anti-*T. gondii* glide-associated protein 45 (TgGAP45) antibodies (1:2000, gifted by Doctor Yonggen Jia, Department of Microbiology and Molecular Medicine, CMU, University of Geneva, Geneva 4, Switzerland) [19] followed by secondary anti-rabbit antibodies coupled to Alexa Fluor 488. After permeabilization with 0.1% Triton X-100 for 30 min, intracellular and adherent external parasites were labeled with anti-GAP45 antibodies followed by secondary anti-rabbit antibodies coupled to Alexa Fluor 594. Data were compiled from three independent experiments. Ten fields were randomly selected in the same pattern (the operator moved the microscope stage without viewing the sample) for all samples, and the number of external and internal parasites was counted in a blinded manner.

### Intracellular growth assay

HFF monolayers were incubated with TgMIC1 polyclonal antibody at 37 °C for 16 h. The TgCtwh3 or TgCtwh6 parasites were allowed to invade HFF cells for 24 h. After washing with PBS three times, the coverslips were fixed with methyl alcohol for 5 min followed by the Wright Giemsa method and observed under a microscope. Fifty PVs were randomly selected, and the number of *T. gondii* tachyzoites in each PV was counted.

### Statistical analysis

Statistical results are shown with values expressed as means ± standard deviation of at least three independent experiments. The statistical significance of differences was performed using one-way ANOVA and *t*-test. Values of *p* < 0.05 were considered statistically significant.

## Results

### Differential mRNA expression of TgMIC1/4/6 in TgCtwh3 and TgCtwh6 strains

**Fig. 1:**
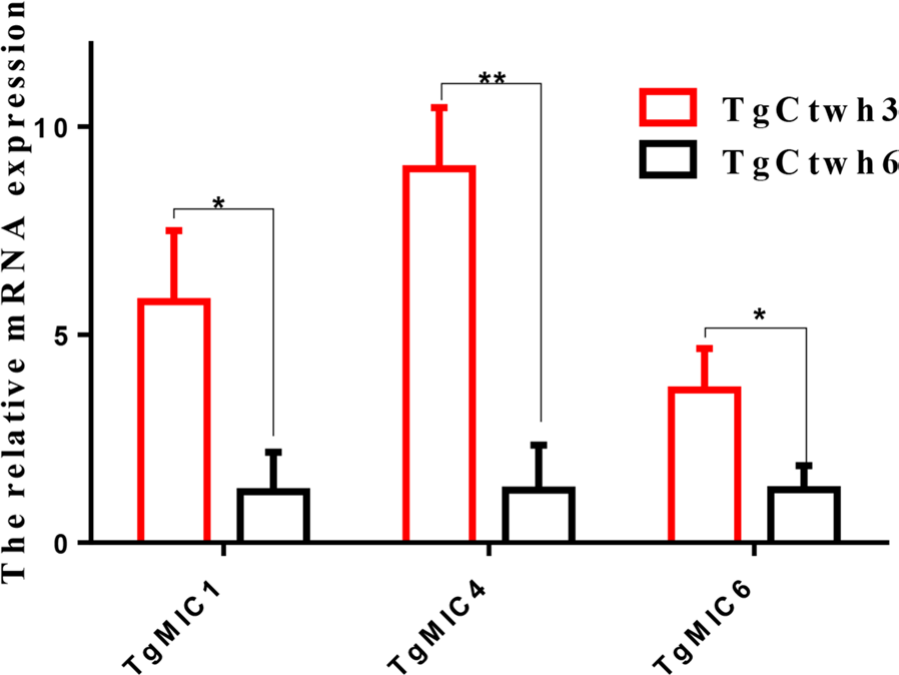
Quantitative real-time PCR analysis of TgMIC1, TgMIC4 and TgMIC6 mRNA expression in TgCtwh3 and TgCtwh6 strains. *p < 0.05, **p < 0.01 versus TgCtwh6 group. Data are represented as mean ± SD for three independent experiments

Virulence genes were identified by comparing and studying the differential gene expression between the TgCtwh3 and TgCtwh6 strains. Quantitative real-time PCR was used to identify the mRNA expression of TgMIC1, TgMIC4 and TgMIC6 in TgCtwh3 and TgCtwh6 strains. Significantly high expressions of TgMIC1 (*t*-test: *t* (4) = 4.047, *p* = 0.015), TgMIC4 (*t*-test: *t* (4) = 7.331, *p* = 0.0018) and TgMIC6 (*t*-test: *t* (4) = 3.609, *p* = 0.022) were observed in the virulent TgCtwh3 compared with the less virulent TgCtwh6 (Fig. 1).

### Codon optimization and efficiency of the TgMIC1 gene

**Fig. 2:**
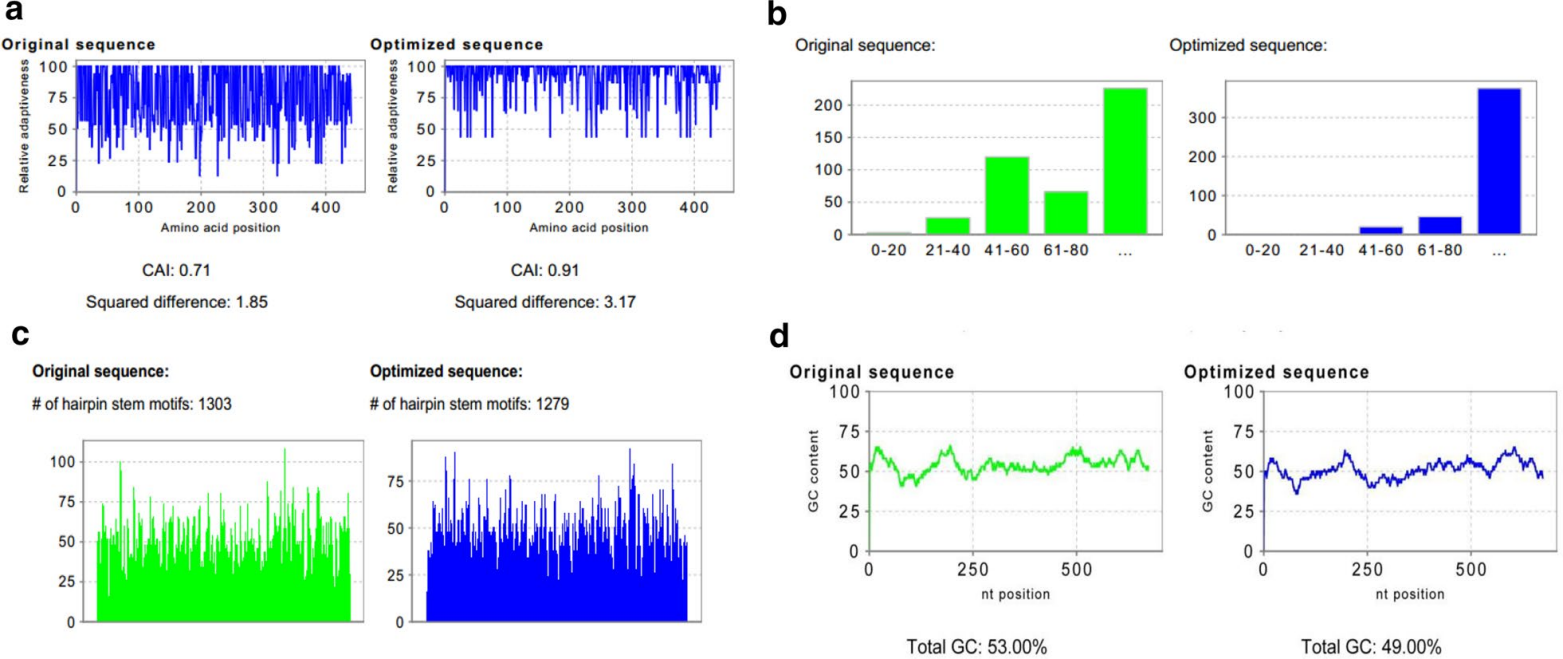
Optimization of TgMIC1 codon sequences. **a** Relative adaptiveness distribution of the original and optimized gene sequences. CAI > 0.9 is regarded as very good in terms of a high gene expression level. **b** The percentage distribution of codons in the original and optimized groups. A value of 100 is set for the codon with the highest usage frequency for a given amino acid in the desired expression organism. **c** Total GC content of the original and optimized genes. **d** Comparison of the mRNA secondary structure between the original and optimized groups

Optigene™ Codon Optimization Analysis Platform was used to optimize multiple important parameters of the TgMIC1 gene to stabilize DNA fragments and in turn increase gene expression efficiency. After optimization, the TgMIC1 Codon Adaption Index (CAI) increased from 0.74 to 0.91, reaching a high gene expression level (Fig. 2a). The frequency of optimal codons (FOP) for TgMIC1 was maintained above 80, which was significantly higher than that of the original sequence (Fig. 2b). The number of secondary structures of mRNA in the TgMIC1 optimized group was slightly lower (Fig. 2c). Moreover, the GC% peaks were removed in a 60-bp window, and the ideal percentage range of GC content was between 30 and 70%. The GC content of the optimized group was lower than that of the previous group (Fig. 2d). The results revealed that the optimized sequence of TgMIC1 was more suitable for the next experiment than the original sequence.

### Expression, purification and identification of the recombinant TgMIC1 protein

**Fig. 3:**
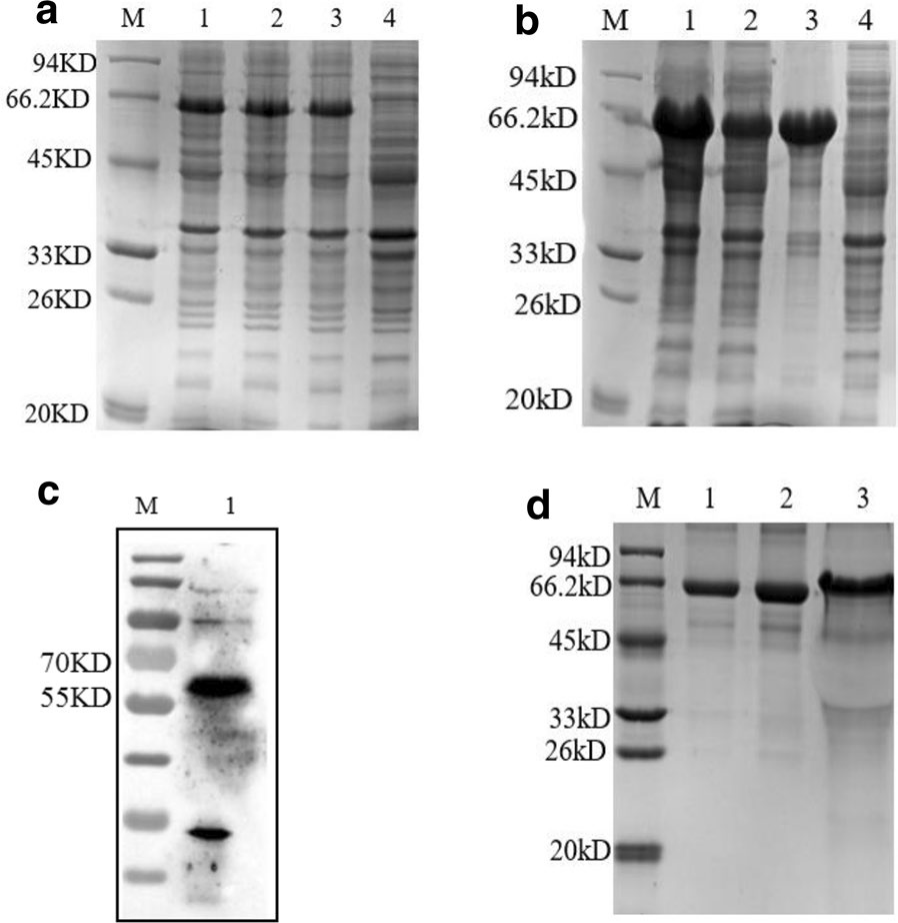
SDS-PAGE and western blot analysis of the rTgMIC1 protein. **a** Expression of pET30a-MIC1/BL21 in small amounts. Lane M: protein marker; lane 1–3: mono-clone picked from conversion plates after induction with IPTG; lane 4: uninduced control. **b** Large amounts of expression detection. Lane M: protein marker; lane 1: proteins in pET30a-MIC1/BL21 after induction with IPTG; lane 2: supernatant of pET30a-MIC1/BL21 bacteria after ultrasonication; lane 3: precipitation of pET30a-MIC1/BL21 bacteria after ultrasonication; lane 4: uninduced control. **c** Western blot analysis of the rTgMIC1. Lane M: protein marker; lane 1: rTgMIC1 detected by HIS monoclonal antibodies. **d** The rTgMIC1 purified by Ni^2+^-affinity chromatography. Lane M: protein marker; lane 1: 0.5 mg/ml BSA; lane 2: 1.0 mg/ml BSA; lane 3: purified rTgMIC1 proteins

The recombinant plasmid pET-30a-MIC1 was successfully constructed. rTgMIC1 was expressed in *Escherichia coli* strain BL21 (Fig. 3a–b) and identified by HIS monoclonal antibodies (Fig. 3c). Western blot and SDS-PAGE analysis indicated that rTgMIC1 had a molecular weight of approximately 60 kDa, which was consistent with the predicted combined sizes of the protein (49 kDa) encoded by the TgMIC1 gene and HIS-tag from the vector. Additionally, the rTgMIC1 protein was purified by Ni^2+^-affinity chromatography (Fig. 3d). Thus, these results suggest that rTgMIC1 was successfully expressed and purified.

### Generation and specific identification of TgMIC1 polyclonal antibody

**Fig. 4:**
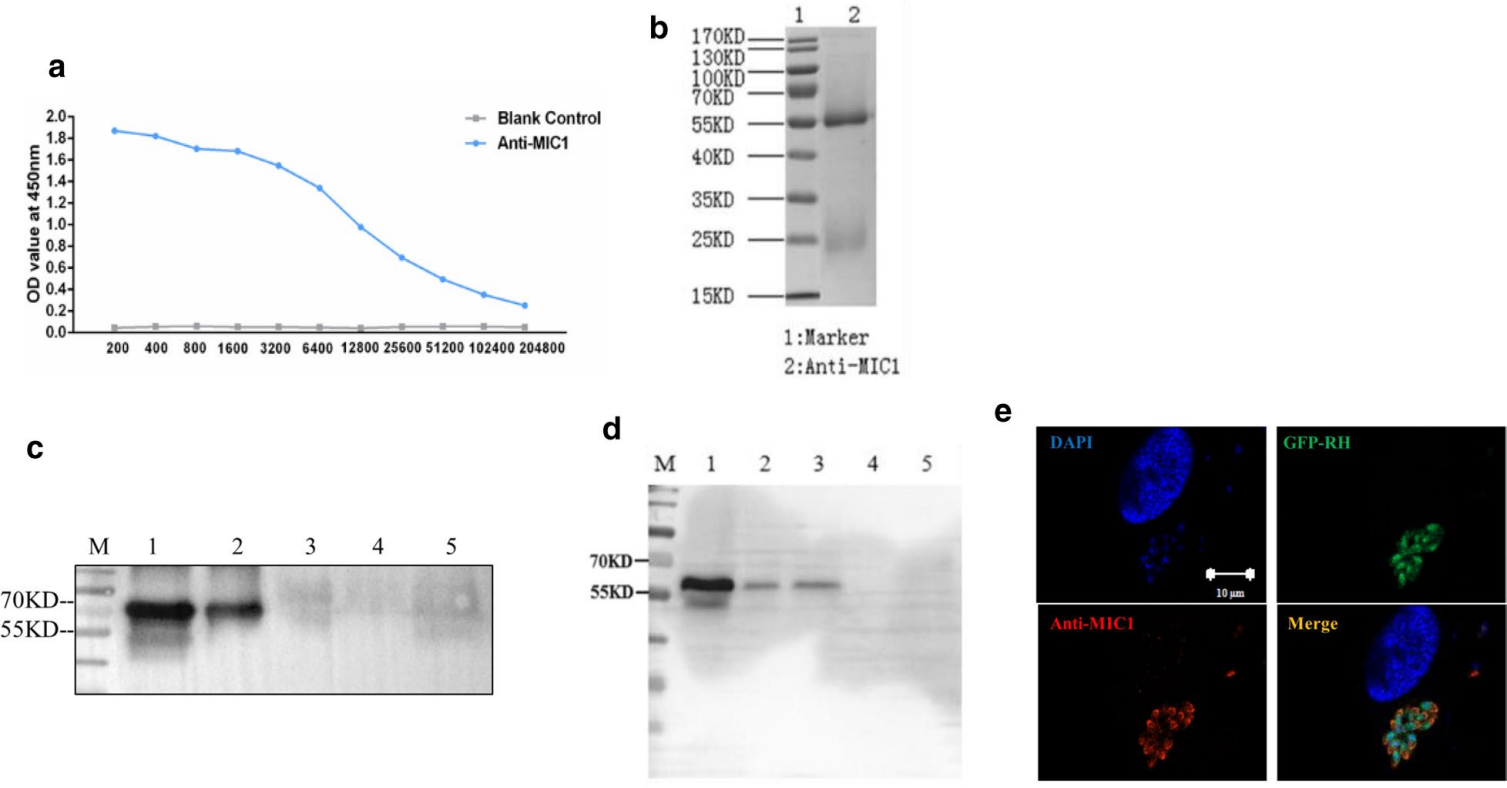
Preparation and specific detection of TgMIC1 polyclonal antibody. **a** The titer of the purified TgMIC1 polyclonal antibody was detected by indirect ELISA. The titer is the dilution corresponding to the minimum OD reading greater than the maximum OD/2. **b** The purity of TgMIC1 polyclonal antibody indicated by SDS-PAGE. Lane M: protein marker; lane 1: heavy and light chains of the purified TgMIC1 polyclonal antibody. **c** Different recombinant bacteria from *Toxoplasma* proteins reacted with the TgMIC1 polyclonal antibody. Lane M: protein marker; lane 1: rTgMIC1; lane 2: pET30a-MIC1 bacteria; lane 3–5: pET30a-MIC4, pET30a-MIC6 and pET28a-ROP18, respectively. **d** The TgMIC1 polyclonal antibody reacted with proteins from different type of *T. gondii* strains and parasites. Lane M: protein marker; lane 1–6: RH, TgCtwh3, TgCtwh6, Plasmodium, and Schistosoma, respectively. **e** Confocal microscopy images showing the localization of TgMIC1 (red) in GFP-RH tachyzoites (green) challenges HFF cells for 24 h. DAPI (blue) was used to stain the nuclei. The scale bars are 10 µm

For further research, the purified rTgMIC1 protein was injected into the New Zealand white rabbit by multiple back injections, and antiserum was collected after the fourth booster immunization. The polyclonal antibody (pAb) against TgMIC1 was purified by Protein A; the titer was approximately 1:12,800, as estimated by ELISA assay (Fig. 4a), with purity > 90%, as indicated by SDS-PAGE analysis (Fig. 4b). The specificity of rTgMIC1 and pET30a-MIC1 was identified by the polyclonal antibodies against TgMIC1 as a clear band of approximately 60 kDa, whereas the other recombinant parasite proteins, pET30a-MIC4, pET30a-MIC6 and pET28a-ROP18, were not identified by the TgMIC1 antibodies (Fig. 4c). Moreover, parasite proteins of different types of *T. gondii* strains, such as RH, TgCtwh3 and TgCtwh6 total proteins, were identified by TgMIC1 antibodies; other parasite proteins, such as *Plasmodium* and *Schistosoma* total protein, were not detected by TgMIC1 antibodies (Fig. 4d). Immunofluorescence with TgMIC1 (red) was performed to determine the localization of TgMIC1 in GFP-RH-infected HFF cells (Fig. 4e). These results indicated that the obtained TgMIC1 antibodies which located in the apex of parasite with high specificity can be used for further experiments.

### Differential protein expression of TgMIC1 in TgCtwh3 and TgCtwh6 strains

**Fig. 5:**
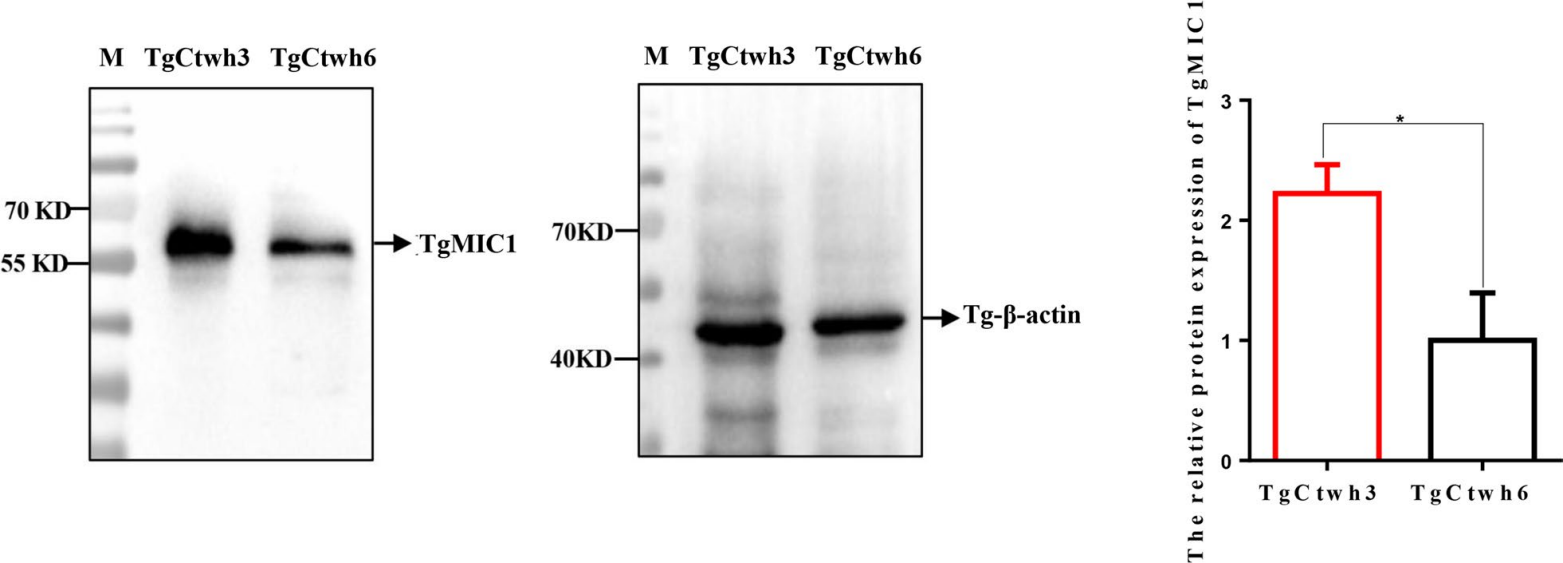
Differential expression of TgMIC1 protein in TgCtwh3 and TgCtwh6 strains. Total proteins extracted from TgCtwh3 and TgCtwh6 tachyzoites were examined for the expression of TgMIC1 by western blot analysis. Tgβ-actin was used as a reference protein to analyze the *T. gondii* protein quantitatively. *p < 0.05 versus TgCtwh6 group. Data are represented as mean ± SD for three independent experiments

Western blot analysis revealed that the relative protein expression of TgMIC1 (*t*-test: *t* (4) = 4.577, *p* = 0.0102) in TgCtwh3 tachyzoites was twofold higher compared to TgCtwh6 tachyzoites (Fig. 5), in agreement with the differential mRNA expression of TgMIC1. Inspired by these results, we hypothesize that differentially expressed TgMIC1 is likely one of virulence regulators between TgCtwh3 and TgCtwh6 strains.

### Evaluation the role of TgMIC1 for parasite invasion and replication

**Fig. 6:**
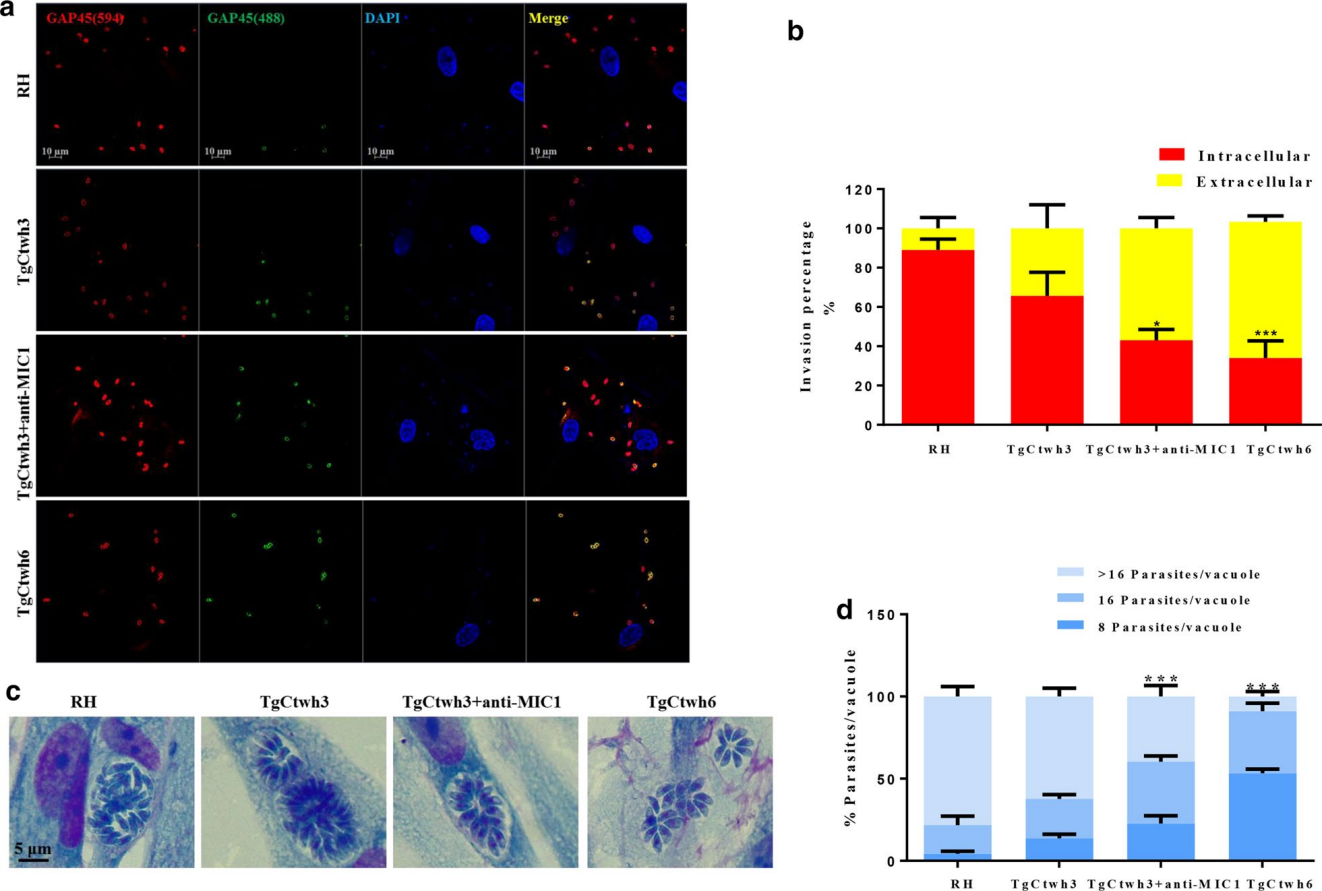
TgMIC1 polyclonal antibody regulates parasite invasion and growth. **a** Representative images of invaded and attached tachyzoites with HFF cells. Infected HFF cells were incubated with TgGAP45 polyclonal antibody and then analyzed by immunofluorescence staining to evaluate the invasion rate of *T. gondii*. Extracellular (yellow) and intracellular (red) tachyzoites in merged pictures; DAPI (blue) was used to stain the nuclei. Scale bar, 10 μm. **b** Histogram of *T. gondii* invasion. The tachyzoites were counted under a microscope (200×). **c** Microscopy showing the growth of parasites in HFF monocytes. Scale bar, 5 μm. **d** The percentage of parasites/vacuole histogram; the tachyzoites were counted under a microscope (400). *p < 0.05, ***p < 0.001 versus TgCtwh3 group. Data are represented as mean ± SD for three independent experiments

From the superimposed picture, red parasites are the parasites that invade the cell, yellow ones are the parasites outside the cell, and the nucleus is blue. The results showed that the invasion efficiency of TgCtwh3, TgCtwh6 and TgCtwh3 plus anti-mic1 was 67%, 40% and 43%, respectively (Fig. 6a–b). The invasion efficiency of TgCtwh3 was higher than that of TgCtwh6 (ANOVA: *F* (3, 16) = 61.13, *p* < 0.001), and the invasion efficiency of TgCtwh3 was reduced by TgMIC1 polyclonal antibody to some extent (ANOVA: *F* (3, 16) = 61.13, *p* = 0.0170). Furthermore, the intracellular replication capability of TgCtwh3 was also attenuated by the TgMIC1 polyclonal antibody compared with the untreated group (ANOVA: *F* (6, 24) = 115.6, *p* < 0.001) (Fig. 6c–d). These results indicate that the level of TgMIC1 affects TgCtwh3 attachment and replication capability in HFF cells.

## Discussion

Much progress has been made toward understanding *T. gondii*'s strain-specific virulence factors and their effects on the host immune response. However, most attention has been focused on the dominant strains in North America and Europe; the information is less clear when considering the dominant strains in other regions, especially in China [20–22]. ROP18/ROP5/ROP17 specifically counteracts murine defense mechanisms by phosphorylating and inactivating immunity-related GTPases (IRGs). Moreover, ROP18 can phosphorylate host activating transcription factor 6β (ATF6β), leading to the proteasomal degradation of ATF6β and thereby interrupting its role in antigen presentation by DCs [23, 24]. Generally, the aforementioned parasite-derived polymorphic effectors represent most of the differences in acute virulence among these three classical lineages. However, this does not seem to explain the differences in toxicity between TgCtwh3 and TgCtwh6 strains; neither genetic variation in exon regions nor differential expression of ROP5 and ROP18 was noted between these two strains (unpublished data). Given its role in altering host gene transcription, ROP16 has been extensively studied as an important virulence-related molecule. Moreover, TgCtwh3 and TgCtwh6 of type Chinese I carry ROP16_I/III_ and GRA15_II_ effectors [25], and there is no significant difference in the virulence of TgCtwh3Δrop16 and TgCtwh3 WT strains in mice [26]. The genotype varies among different strains of *T. gondii* and results in divergent resistance to host defense mechanisms, suggesting that type I Chinese strains have unique virulence characteristics and pathogenesis.

Recent advances in the field of *T. gondii* research have revealed that microneme secretion is regulated by phosphatidic acid, cyclic guanosine monophosphate and calcium [27, 28]. These TgMIC proteins cooperate with actomyosin system control tachyzoite invasion and egress from infected cells [13, 29]. Among the released proteins, TgMIC1, TgMIC4 and TgMIC6 form a complex. This resultant complex, together with other *T. gondii* proteins, acts on the adhesion and invasion of host cells and promotes the virulence of *T. gondii* [13, 30]. Our results demonstrated that the mRNA level of TgMIC1 in TgCtwh3 is apparently higher than in TgCtwh6. Given the remarkable role of TgMIC1, we developed rTgMIC1 protein and pAbs against it to identify its protein expression in these two strains. Furthermore, the native gene of TgMIC1 has several features which may lead to poor expression; hence, we strategically optimized the underlying DNA sequence of TgMIC1 to improve its mRNA stability and recombinant protein expression in *E. coli*. Compared with that of the actual codon frequency, the squared difference of the preferred codon frequency changed from 1.85 to 3.17. The GC content throughout the sequence was homogenized to increase the half-life of the mRNA. Moreover, the mRNA secondary structure has been reduced, which improved the translation efficiency. These modifications helped us to obtain the high yield and high concentration of TgMIC1 protein. In our study, the produced pAbs could recognize not only the recombinant TgMIC1 but also the natural TgMIC1 protein in *T. gondii* but not those in *Plasmodium* and *Schistosoma*.

Factors such as invasiveness, intracellular replication capability and the strength and characteristics of the induced immune response in host cells combine to determine *T. gondii*'s virulence. A previous study reported that the invasion by TgMIC1ko parasites was reduced by half compared to wild type [31]. An important observation in the present study is that the protein level of TgMIC1 in TgCtwh3 was significantly higher than that in TgCtwh6, which may cause the difference invasiveness or replication capability of *T. gondii* to host cells. Consistent with our hypothesis, the two-color and experimental analysis of *T. gondii*-infected HFF cells revealed that elimination of TgMIC1 protein by adding exogenous polyclonal antibodies hinders the invasiveness and intracellular replication capability of TgCtwh3. Invasion of host cells by *T. gondii* involves carbohydrate recognition [32, 33], and TgMIC1 has lectin domains that recognize oligosaccharides with sialic acid in the terminal position [34]. rTgMIC1 stimulates dendritic cells and macrophages produce proinflammatory cytokines via engaging TLR2 and TLR4 [17]. TgMIC1 was highly expressed in the Chinese 1 genotype *T. gondii* high virulent strain TgCtwh3, which enhanced its invasion ability and, in contrast, may contribute to *T. gondii* proliferation through participation in cellular immunity. Further experiments with TgCtwh3-MIC1 ko and TgCtwh3-MIC1 ko + MIC1 strains constructed using CRISPR/CAS9 technology are needed to study the TgMIC1 function in TgCtwh3 and TgCtwh6 strains.

## Conclusions

Taken together, we have successfully developed rTgMIC1 and its pAb. These outcomes can be applied to many experiments, from western blotting and immunofluorescence to *in vivo* function analysis. Furthermore, our work clearly shows the effect of differences in TgMIC1 levels on Chinese I *Toxoplasma* intracellular invasion and proliferation; this represents an important step toward our understanding of how TgCtwh3 and TgCtwh6 vary in their virulence in mice.

## Data Availability

The datasets used and/or analyzed during the current study are available from the corresponding author on reasonable request.
